# Inhibition of transforming growth factor-β in osteoarthritis. Discrepancy with reduced TGFβ signaling in normal joints

**DOI:** 10.1016/j.ocarto.2022.100238

**Published:** 2022-02-04

**Authors:** Peter M. van der Kraan

**Affiliations:** Radboud University Medical Center, Experimental Rheumatology, Interdisciplinary Consortium for Clinical Movement Sciences and Technology. PO Box 9101, 6500HB, Nijmegen, the Netherlands

**Keywords:** Transforming growth factor-beta, Experimental osteoarthritis, Cartilage, Animal models

## Abstract

**Objective:**

Transforming growth factor-β (TGFβ) is a pleiotropic cytokine that is central in the regulation of joint health and disease. Inhibition of TGFβ activity/signaling in experimental osteoarthritis (OA) has been performed to modulate OA severity and progression. In this narrative review we discuss the potential reasons for the variable results of TGFβ inhibition in these models.

**Design:**

A literature study was performed using the search terms; experimental osteoarthritis and TGFβ. Papers were selected that describe the effect TGFβ activity/signaling inhibition on experimental OA. Based on the selected papers a narrative review has been written about the potential therapeutic role of TGFβ inhibition in OA and potential causes for its variable effects are discussed.

**Results:**

Inhibition of TGFβ activity in experimental models of OA does not result in either straightforward protection or deleterious effects. More than half of the studies (13/19), but not all, report that inhibition of TGFβ in experimental OA reduces OA severity. This is in contrast with the protective role of TGFβ in healthy joints.

**Conclusions:**

The effect of TGFβ inhibition on joint damage in experimental OA is variable. Most likely this is a consequence of the changing function of TGFβ in normal and OA joints. As a result, the overall outcome of TGFβ modulation in OA will be unpredictable. To develop OA therapies based on modulation of TGFβ activity specific protective and damaging signaling routes should be identified and tools developed to block the damaging ones.

## 1 Introduction

Osteoarthritis (OA) is the most common joint disease, and is increasing in prevalence in the Western world owing to lifestyle and ageing of the population [1]. The etiology of OA is multifactorial, including heredity factors, obesity, ageing and joint injury [2]. The primary characteristics of OA are destruction of articular cartilage, synovial inflammation and fibrosis, osteophyte formation and subchondral bone alterations. OA is progressive and can result in loss of joint function.

Transforming growth factor-β (TGFβ) is a pleiotropic cytokine that is important in the regulation of joint homeostasis and disease. In articular cartilage, TGFβ signalling is induced by loading and has an important function in maintaining the differentiated phenotype of articular chondrocytes and dampening of inflammation [3]. Synovial fluid concentrations of active TGFβ differ greatly between healthy and osteoarthritic joints, being low in healthy joints and raised in osteoarthritic joints, leading to the activation of different signalling pathways in joint cells [4].

Functional TGFβ is a 25 kD dimer that signals via heteromeric complexes of transmembrane serine/threonine type I and type II receptors, most frequently tetramers composed of two type I and two type II receptors [5]. Receptor binding and signaling efficiency are modulated by betaglycan and endoglin. These are membrane-bound co-receptors of the TGFβ family that selectively recognize and potentiate the functions of their ligands [6].

The canonical signalling route of TGFβ is via the broadly expressed type I receptor activin-like kinase 5 (ALK5), but in chondrocytes and other cell types additional ALKs, such as ALK1 (ALK2, ALK3), can be involved [[7], [8], [9]]. Upon type I receptor activation, intracellular signaling is initiated by C-terminal phosphorylation of receptor-regulated (R)-SMAD proteins. Whereas ALK5 stimulates phosphorylation of SMAD2 and SMAD3, ALK1 (ALK2, ALK3) mediates the activation of SMAD1, SMAD5 and SMAD8 (also known as SMAD9). Phosphorylated R-SMADs form heteromeric complexes (trimers) with SMAD4, the common mediator SMAD (co-SMAD); the trimers are made up of two R-SMADs and one SMAD4. These heteromeric complexes accumulate in the nucleus where they, together with co-activators and repressors, control transcriptional responses. Different heteromeric complexes control expression of different genes.

In addition to the above described canonical (SMAD-dependent) signaling pathways alternative pathways (SMAD-independent) are described. Recently, a review on non-canonical pathways in TGFβ has been published [10]. TGFβ has been shown to activate various non-canonical pathways including mitogen activated protein (MAP) kinases (ERK, p38 and JNK), phosphatidylinositol-3-kinase (PI3K) and Rho-like GTPases, and Janus kinases (JAK)-signal transducer and activator of transcription proteins (STAT). Unrelated to their kinase activity, type I receptors can function as dockings stations to recruit, assemble and activate signaling proteins that act independent of SMAD signaling but that can also alter SMAD signaling by modification of the SMAD protein in its linker domain [11,12].

In articular cartilage, TGFβ is stored in latent form in large amounts (∼300 ​ng/ml) and compressive loading rapidly activates TGFβ signalling in cartilage explants, implying that under normal physiological conditions (regular loading) articular cartilage has a basic level of TGFβ signalling and expression of TGFβ-regulated genes [13,14]. Mutations in the TGFβ signaling cascade, for instance in Smad3, a central molecule in TGF-β signalling, result in cartilage degeneration. Mutations in Smad3 have been found in patients with early-onset OA while genetic variations in the Smad3 gene are associated with knee and hip OA [15,16]. Deletion of Tgfbr2, encoding for the type II TGF-β receptor, Smad3 or ALK-5 in articular chondrocytes in mice consistently leads to an OA-like phenotype [[17], [18], [19], [20], [21], [22]]. TGFβ inhibits chondrocyte hypertrophy and stimulates expression of Prg4, which encodes for lubricin, a vital molecule for lessening joint friction [23,24]. Furthermore, it limits inflammatory pathways, for instance IL-6 signaling [25,26]. These findings show that active TGFβ signaling is an absolutely vital event in sustaining cartilage homeostasis.

Although loss of TGFβ signalling is deleterious for healthy articular cartilage, in OA joints another situation can occur. Synovial fluid and tissues of OA joints will contain elevated levels of active TGFβ compared to healthy joints. Elevated TGFβ levels most likely contribute in OA joints to synovial fibrosis, osteophyte formation, subchondral bone changes and detrimental changes in chondrocyte phenotype and function. Considering the context dependent function of TGFβ in normal and OA joints the outcome of either inhibition or supplementation of TGFβ in experimental OA is hard to predict. In this article an overview will be given on the effect of TGFβ signaling modulation in experimental OA models and the consequences for the use of TGFβ-dependent OA therapy will be debated.

## 2 Change in osteoarthritis severity by modulation of TGFβ signaling in animals models

Inhibition of TGFβ activity during OA in animal models results in many instances in inhibition of OA severity and progression (Table 1). One of the first studies that showed that inhibition of TGFβ signaling in a model of experimental OA lessens OA severity is by Zhen et al. [27]. They show in mice that blocking TGFβ signaling, using the ALK5 inhibitor SB-505124 (1 ​mg/kg), inhibited OA severity as indicated by subchondral bone changes and proteoglycan loss. However, high concentrations of SB-505124 (2,5 and 5 ​mg/kg) induced proteoglycan loss in articular cartilage, demonstrating that robust blocking of TGFβ activity in articular cartilage leads to harm. Their findings indicate that TGFβ can play a differential role in experimental OA, increasing OA severity via its action on subchondral bone as opposed to its anabolic effect on articular cartilage [27]. A deleterious effect of TGFβ on OA development via its action on subchondral bone has also been demonstrated using the TGFβ neutralizing antibody 1D11 [28].

**Table 1:** Change of OA severity by TGF-β activity modulation in experimental OA. (TMJ-temporomandibular joint).

Species	Reduction of OA severity	TGF-β inhibitor	references
	OA model, joint		
Mouse	Anterior cruciate ligament transection, stifle	SB-505124	[27]
Mouse	Ageing, TMJ	SB-505124	[29]
Mouse	Camurati–Engelmann TGF-β1 mutation, TMJ	SB-505124	[29]
Mouse	Destabilization medial meniscus, stifle	losartan	[31]
Mouse	Col11a1 mutation, stifle, TMJ	losartan	[32]
Mouse	Anterior cruciate ligament transection, stifle	halofuginone	[33,34]
Mouse	Anterior cruciate ligament transection, stifle	halofuginone	[65]
Mouse	Anterior cruciate ligament transection, stifle	Antibody 1D11	[28]
Rat	Abnormal dental occlusion, TMJ	SB-505124	[29]
Rat	Anterior cruciate ligament transection, stifle	halofuginone	[65]
Rat	Papain injection, stifle	SB-505124	[35]
Rat	Anterior cruciate ligament transection, stifle	SB-505124	[36]
**Species**	**Increase in OA severity**	**TGF-β modulator**	**references**
	**OA model, joint**		
Mouse	Papain-induced, stifle joint	scavenging soluble TGF-β –RII	[37]
Rabbit	Disc perforation, TMJ	TGF-beta	[39]
Mouse	Destabilization medial meniscus, stifle	BSHXF	[22,38]
Mouse	TGFβ-RII knock out	BSHXF	[22,38]

In a study on temporomandibular (TMJ) joint OA, using three different models, inhibition of the ALK5 with SB-505124 attenuated OA progression in these models [29]. Conditional removal of *Tgfbr2* in mature condylar cartilage significantly delayed the progression of the cartilage degeneration induced by a partial discectomy. Increase in the expression and activity of TGFβ signaling has detrimental effect on mature condylar cartilages [30]. Therefore, inhibition of TGFβ signaling may be able to protect condylar cartilages from being degraded in mature TMJ joints.

Eight week old wild-type mice subjected to stifle joint destabilization by dissection medial meniscus model (DMM) and treated with the Tgfbr2 inhibitor losartan for 8 weeks showed delay of articular cartilage degeneration [31]. Another murine OA model, displaying a col11a1 mutation, treated with losartan showed less cartilage degeneration than non-treated mice [32]. However, it has to be kept in mind that losartan is an angiotensin II type 1 receptor blocker and not a specific TGFβ activity inhibitor. The same holds true for the TGFβ inhibitor halofuginone that also has been shown to attenuate OA severity in experimental models in mice [33,34]. Application of SB-505124 in a rat OA model induced by papain led to reduced cartilage damage, and expression levels of TGFβ1, Smad2, Smad3 and ALK5 were higher in the control OA group than those in the inhibitor group. The rats in the inhibitor group had also decreased expressions of phospho-Smad2/3 and ALK5, as well as decreased numbers of type X collagen and Osterix positive cells when compared with the OA model group. These findings indicated that the degree of cartilage damage among rats with papain-induced OA was improved by inhibiting the TGFβ signaling pathway [35]. In a rat anterior cruciate ligament transection (ACTL) model, interruption of TGFβ signalling (SB-505124) markedly attenuated articular cartilage degradation indicating that overexpressed TGFβ is involved in articular cartilage degeneration and OA-like changes in this model [36]. All the above mentioned studies indicate that blocking TGFβ limits OA progression in experimental OA in small animal models.

It has been without doubt shown that normal TGFβ signaling has an essential role in cartilage maintenance. The effect of inhibition of TGFβ signaling during OA appears to be more complex. Above studies describe that during OA TGFβ has a deleterious effect on joint integrity but also studies have been published that indicate the opposite (Table 1). We determined the role of endogenous TGFβ on osteophyte formation and articular cartilage damage in a papain-induced mouse model of osteoarthritis [37], TGFβ activity was blocked via a scavenging soluble TGF-β-RII. Inhibition of endogenous TGFβ prevented osteophyte formation but enhanced articular cartilage proteoglycan loss and reduced the thickness of articular cartilage. Wang et al. investigated the effects of Bushenhuoxue formula (BSHXF) in DMM-induced OA mouse model [22,38]. Elevated TGFBR2 and pSMAD2, were revealed in DMM-induced mice treated with BSHXF. BSHXF decreased cartilage degradation and chondrocyte apoptosis, reduced OARSI score while increasing cartilage area and thickness, proteoglycan and type II collagen content. In addition, clear improvement in articular cartilage structure was observed in *Tgfbr2* knock out mice after BSHXF treatment, related with up-regulated pSMAD2 expression levels in articular cartilage. However, it has to be kept in mind that BSHXF might have additional effects in OA joints than activation of TGFβ signalling explaining the observed effects. In another study, injection of TGFβ on articular cartilage in mice with osteoarthritic TMJ OA prevented OA-like changes. A significantly greater expression of aggrecan and collagen type II was found in the experimental compared with the control joints [39].

## 3 Correlation of TGFβ levels and human OA severity

No human studies have been reported investigating the effect of blocking TGFβ activity in OA. Only the effect of injecting genetically engineered chondrocytes expressing TGFβ1 into the knees of patients with OA (phase II trial) has been published [40]. However, a correlation between OA severity and serum TGFβ concentrations have been reported, although this has not been confirmed by all studies published. A recent study of Hsueh et al. demonstrated that expression levels of TGFβ1 in serum were significantly associated with severity in human radiographic knee OA. Baseline TGFβ1 concentrations, along with radiographic knee OA severity scores, were predictive of knee OA progression [41]. Another study by He et al. shows that TGFβ1 serum levels in knee OA were significantly higher compared to controls. Average serum level of TGFβ1 was positively associated with Kellgren-Lawrence (K-L) grades [42]. A Greek study showed that not TGFβ1, but TGFβ2 and -3 serum protein levels were significantly higher in knee OA patients compared to controls. Additionally, TGFβ2 and -3 were strongly positively correlated to radiographic K-L grading of the disease. Moreover, TGFβ2 correlated positively with the WOMAC scale [43]. Punzi et al. showed that TGFβ levels in OA joints (synovial fluid) in which the presence of crystals was demonstrated was much higher than in crystal negative joints, respectively 66,1 and 18,2 ​ng/ml [44]. Two studies of Nelson et al. did not demonstrate a significant correlation between serum TGFβ levels and OA. Patients with osteophytes had high TGFβ levels but this association was not significant. There were no significant differences in mean TGFβ levels by severity of knee or hip OA [45,46]. Concluding, it appears that several studies indicate a correlation between serum TGFβ concentrations and OA severity. That this is not found in all studies which could be due to different methods to measure TGFβ levels and variable patient populations. Moreover, TGFβ concentrations are notoriously difficult to measure reliable due to its sticking properties and can depend upon age, smoking, gender and comorbidities [47]. Overall, there appears to be an association between serum TGFβ levels but this link does not necessarily indicates a causal relationship, elevated TGFβ levels can be the either the source or the reflection of joint damage. Only effective blocking studies in well-defined patients groups can solve this enigma.

## 4 Possible reasons for differential effects of TGFβ modulation in experimental OA

The studies described above show that in most studies blocking TGFβ activity in animal models of OA results in reduced OA severity, but not all. These differential results, in combination with the observation that lack of TGFβ signaling in a normal joint unavoidably leads to OA, clearly point out to a circumstantial role of TGFβ in the articular joint. What could be the root for these different roles of TGFβ?

## 5 Different concentrations of active TGFβ in normal and OA joints

Under physiological circumstances, the concentration of active TGFβ in joint tissues and synovial fluid is very low. In normal articular cartilage, compressive loading activates TGFβ, but at a low level and temporarily [14]. However in OA, it can be expected that the pathological changes that take place in the joint will change this situation. Joint cells, such as macrophages and fibroblasts, can produce and activate TGFβ [48,49]. Furthermore, it has been shown that inflammation results in increased levels of active TGFβ [50]. Given that articular cartilage contains a reservoir of TGFβ bound to the matrix, cartilage degradation is expected to release TGFβ. The high level of proteolytic activity in osteoarthritic joints will further contribute to TGFβ activation.

Regrettably, most studies measuring synovial fluid TGFβ concentrations have only reported total TGFβ levels, and as mentioned above TGFβ concentrations are notoriously difficult to measure, which makes it hard to draw a reliable conclusion about active TGFβ levels in situ. Fava et al. measured TGFβ activity in the synovial fluid of OA patients using a radioreceptor and a bioassay [4]. The mean level of active TGFβ was ∼4 ​ng/ml (overall estimation 5–10 ​ng/ml active TGFβ in OA joints), whereas in a patient with avascular necrosis no active TGFβ could be measured. Branco de Sousa reported higher TGFβ levels in synovial fluid of knee OA joints than from normal controls [51]. Fang et al. reported that in normal TMJ no TGFβ could be detected while in TMJ with OA it was easily measurable [52]. Moreover, there is evidence that TGFβ is proteolytically activated in synovial fluid [53]. These results indicate that active TGFβ levels will be very low in normal articular joints and elevated in joint diseases such as OA.

**Fig. 1:**
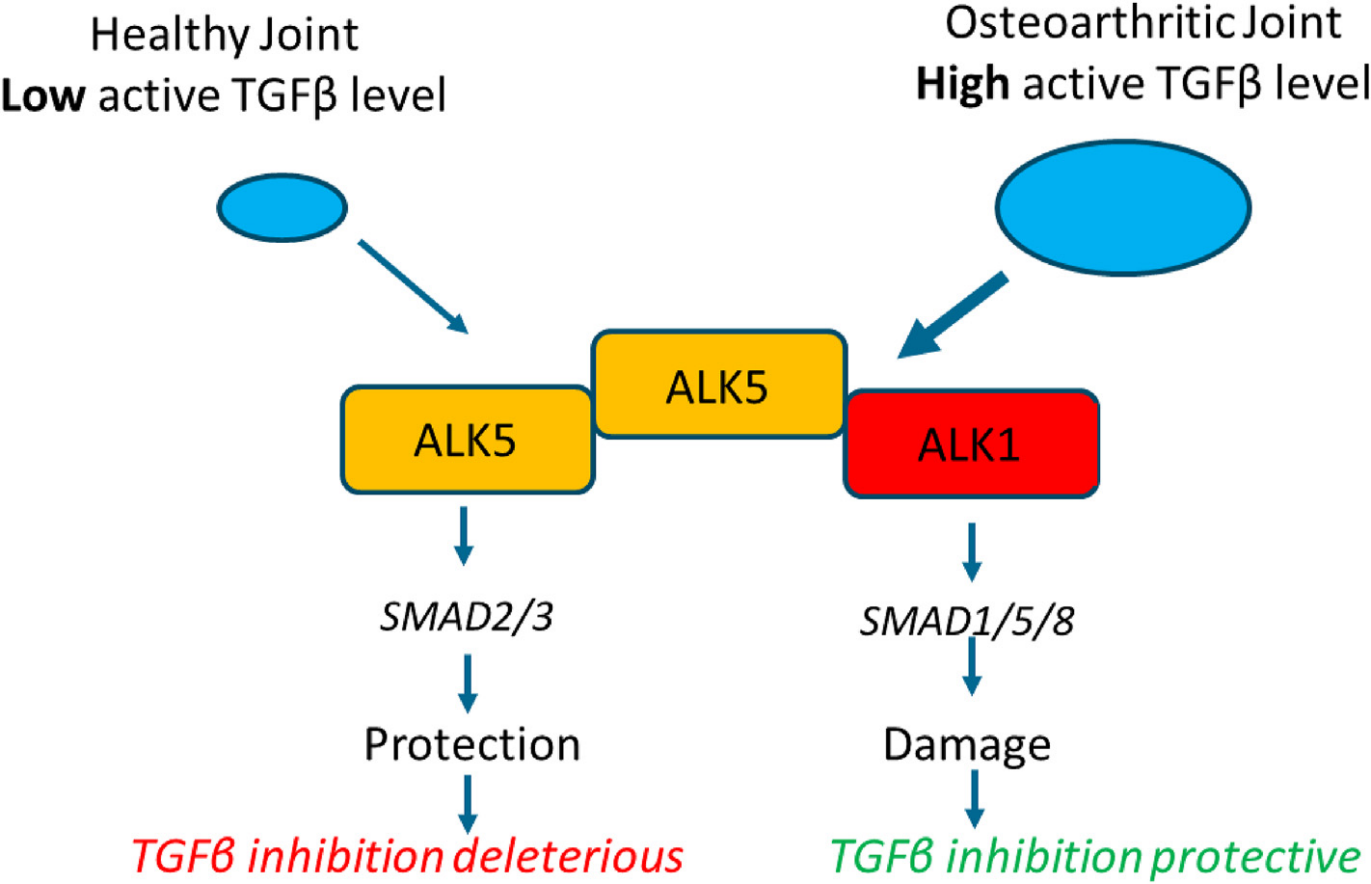
The function of TGFβ is context dependent. TGFβ signalling routes are dependent on active TGFβ concentrations and these concentrations are low in healthy joints and elevated in OA joints.

The consequence of different TGFβ concentrations in healthy and OA joints is activation of differential intracellular pathways. In a normal joint, with low concentrations of active TGFβ, the SMAD2–SMAD3 pathway will be preferentially activated, whereas in a joint with high concentrations the SMAD1–SMAD5–SMAD8 route will be favored (Fig. 1) [54,55]. It has been shown that the Smad2-Smad3 pathway is cartilage protective while SMAD1–SMAD5–SMAD8 signaling promotes chondrocyte hypertrophy and cartilage damage [56]. Moreover, we have found that crypto is overexpressed in OA cartilage and participates in a TGFβ-ALK1-Cripto receptor complex in the plasma membrane, thereby inducing catabolic SMAD1–SMAD5–SMAD8 signaling in chondrocytes [57]. Therefore, blocking low TGFβ concentrations in a healthy joint will lead to joint damage while blocking TGFβ in an OA joint with high TGFβ levels can be anticipated to be protective, as has been shown in most studies of experimental OA.

Besides changes in canonical signaling also disease dependent alterations in non-canonical can play a role. Altered non-canonical signaling can change the function of TGFβ in osteoarthritis. An important factor might be the presence and the magnitude of inflammation in the joint. Inflammation has the potential to modulate the role of TGFβ in the joint by its own effects or by altering SMAD signaling by modulation of the SMAD linker domain [12].

The differential action of TGFβ in a healthy and an OA joint poses a problem for anti-TGFβ treatment. Systemic inhibition will have differential effects on different joints, depending on its disease status and local TGFβ concentrations. As a result, local modulation of TGFβ activity should be the preferred action to treat OA joints. Though, even within a joint TGFβ might have other effects on relatively normal cartilage and severely diseases tissue depending on the local active TGFβ levels.

## 6 Effectivity of anti-TGFβ inhibition and timing of treatment

A factor that also might affect the outcome of treatment is effectivity of anti-TGFβ inhibition. The levels of active TGFβ that remain in the joint will determine what will be the final outcome. Partial inhibition of elevated TGFβ levels can be expected to protect the joint while effective blocking, reducing the active levels to (nearly) zero, could be damaging to the joint. Furthermore, the active concentrations will not be constant but fluctuate depending on the momentary inhibition of active TGFβ.

Another aspect that might modulate the outcome of anti-TGFβ treatment on a theoretical base is the timing of the administration of anti-TGFβ. Is a therapeutic or preventive regime used? In a preventive regime, TGFβ will be blocked from the moment of OA induction while a therapeutic regime will start when structural OA changes are developing. In the study of Scharstuhl, finding a deleterious effect of TGFβ inhibition, blocking TGFβ activity was started before OA induction [37]. BSHX formula was orally administered once a day starting the day after surgery [38]. In a study of Man et al. extra -TGFβ was provided four weeks after TMJ operation. Based on above reasoning at might be expected that OA severity should be increased. However, this is a non-standard OA model based on disc perforation and might more be a model of cartilage repair than of OA. Other reported studies show that inhibition of TGFβ results in less joint damage. Not all studies clearly describe the dosing regimen, but TGFβ inhibition starting right after and until 4 weeks after OA induction are applied, and all these studies show reduced severity when TGFβ activity is blocked (Table 1). Timing does not be an important aspect to determine outcome of TGFβ activity modulation.

## 7 OA models, animal age and target tissues

Different models of experimental OA are used to test the effect of TGFβ inhibition on joint pathology. All reported studies are in small animal models (mouse, rat and rabbit) in stifle joint (knee) or TMJ, and perform chemically-induced or surgically-induced OA or models based on genetic modification or spontaneous mutations. All these models represent a different aspect of human OA and differ in their translatability to human disease. None of the model covers the whole spectrum of human OA. Nonetheless the variability of these models, most studies report a positive effect of TGFβ inhibition on OA severity. This indicates that in the majority of investigations TGFβ has a similar role in the OA disease process in these experimental models.

Responses to TGFβ are age-related and studies in old mice and cows have revealed an age-related decrease in ALK5 expression and a subsequent increase in the ALK1:ALK5 ratio [20,[58], [59], [60]]. Recently we have found that TGFβ induces IL-6 expression in chondrocytes but blocks its action by down-regulating the expression of IL-6 receptor on chondrocytes. This effect is mediated through the ALK-5 receptor and is lost upon ageing, rendering old cartilage sensitive to IL-6-induced damage [25,61]. Whether inhibition of TGFβ has a different effect in old animals than in young animals is not known since comparative studies have not been carried out. On theoretical grounds it can be anticipated that blocking TGFβ in old animals will even have more effect on OA severity than in young animals, since deleterious pathways predominate in old individuals. This is confirmed by the study of van Beuningen et al. who showed that inhibition of interleukin-1-induced proteoglycan synthesis inhibition by TGFβ was more effective in young than in old mice [62].

The outcome of the studies will also depend on the target tissue and the OA scoring system used. Inhibition of TGFβ can have different effects on different joint tissues, for instance articular cartilage or subchondral bone. It has been shown that robust inhibition of TGFβ activity (SB-505124) improved subchondral bone structure but induced proteoglycan loss in articular cartilage [27]. Local TGFβ concentrations, but mainly the role of TGFβ on specific cells types, will determine what will be the effect of TGFβ blockade. For instance, when osteophytes or subchondral bone are a major determinant in the scoring system applied, inhibition of TGFβ will have a clear inhibitory effect since TGFβ is a major driver of osteophyte formation and subchondral bone remodeling [27,63,64].

## 8 Concluding remarks and consequences for therapy

The most striking observation is that inhibition of TGFβ activity/signaling in a normal joint results in joint damage while in most studies, but not all, inhibition of TGFβ in experimental models of OA reduces OA severity. Development of a therapy based on the observed reduction of OA severity by blocking TGFβ activity could be attractive but is complicated by the effect that TGFβ will have on normal cartilage in non-OA affected joints or even within the OA affected joint itself. This makes simple inhibition of TGFβ activity not the preferred option. A better alternative is the development of therapeutic tools that can separate the different TGFβ signaling pathways, blocking the deleterious pathways and concomitantly preserving or stimulating the protective pathways. This means blocking specific receptor complexes or intracellular pathways, a challenging but not impossible option.

## Declarations

I have no competing interests with regard to his manuscript. The manuscript is designed and drafted by the first and sole author.
